# A stumbling block in pancreatic cancer treatment: drug resistance signaling networks

**DOI:** 10.3389/fcell.2024.1462808

**Published:** 2025-01-13

**Authors:** Jinming Liu, Biao Zhang, Bingqian Huang, Kexin Zhang, Fujia Guo, Zhizhou Wang, Dong Shang

**Affiliations:** ^1^ Department of General Surgery, Pancreas and Biliary Center, The First Affiliated Hospital of Dalian Medical University, Dalian, China; ^2^ Key Laboratory of Clinical Cancer Pharmacology and Toxicology Research of Zhejiang Province, Department of Clinical Pharmacy, Affiliated Hangzhou First People’s Hospital, Westlake University, Hangzhou, China; ^3^ Central Laboratory, The First Affiliated Hospital of Dalian Medical University, Dalian, China; ^4^ Institute (College) of Integrative Medicine, Dalian Medical University, Dalian, China

**Keywords:** pancreatic cancer, signaling network, drug resistance, matrix, gemcitabine

## Abstract

The primary node molecules in the cell signaling network in cancer tissues are maladjusted and mutated in comparison to normal tissues, which promotes the occurrence and progression of cancer. Pancreatic cancer (PC) is a highly fatal cancer with increasing incidence and low five-year survival rates. Currently, there are several therapies that target cell signaling networks in PC. However, PC is a “cold tumor” with a unique immunosuppressive tumor microenvironment (poor effector T cell infiltration, low antigen specificity), and targeting a single gene or pathway is basically ineffective in clinical practice. Targeted matrix therapy, targeted metabolic therapy, targeted mutant gene therapy, immunosuppressive therapy, cancer vaccines, and other emerging therapies have shown great therapeutic potential, but results have been disappointing. Therefore, we summarize the identified and potential drug-resistant cell signaling networks aimed at overcoming barriers to existing PC therapies.

## 1 Introduction

With a five-year survival rate of approximately 12%, pancreatic cancer (PC) is one of the deadliest and worst prognosis cancers (Siegel et al., 2023). Although the incidence of PC is relatively low, it significantly increases the risk of cancer-related death, as evidenced by a mortality-to-incidence ratio of 94% (Bray et al., 2018; Sung et al., 2021). Pancreatic ductal adenocarcinoma (PDAC), which is typified by aggressiveness, delayed diagnosis, and a dismal prognosis, is the primary kind of PC (Skinhoj, 1999).

The occurrence of PC depends on the gradual accumulation of driver mutations, such as the oncogene KRAS and the anti-oncogenes CDKN2A, P53, and SMAD4 (Cicenas et al., 2017). These molecular level changes are accompanied by histological changes (Hayashi et al., 2021). Pancreatic intraepithelial neoplasia (PanIN) and intraductal mucinous tumor (IPMN) gradually increased in grade and evolved into PC with the change of histological morphology (Raghavan et al., 2021). As PC progresses, the surrounding tumor microenvironment (TME) is altered (Hirasawa et al., 1998). Normal tissue can respond to injury through connective tissue components, blood vessels, immune cells, etc., to achieve the “wound healing effect”. The matrix of the PC that is changed, which cannot cope with the injury, and even becomes resistant to the treatment drugs, is called “wound healing gone wrong” (Ho et al., 2020). Unlike other solid tumors, PCs have a particularly prominent mesenchymal compartment within the stroma, which usually accounts for most of the tumor volume. This stromal compartment includes cancer-associated fibroblasts (CAFs), extracellular matrix (ECM) components, immune cells, and neural and endothelial cells (Sherman and Beatty, 2023). Alterations in PC stroma are strongly associated with primary and acquired resistance to therapy, not only resisting cytotoxic chemotherapy but also hindering targeted and immunomodulatory therapies (Vennin et al., 2018; Peran et al., 2021).

At present, many therapies targeting the stroma, mutated genes, and metabolism of PC have emerged, as well as immunosuppressive therapies and cancer vaccines, all of which have great therapeutic potential (Hosein et al., 2020; Bannoura et al., 2021; Huang X. et al., 2022; Micevic et al., 2023). But it still falls short of expectations. Therefore, in this review, we summarize the resistant cell signaling networks of existing therapies, aiming to overcome existing therapeutic challenges.

## 2 Drug resistance signaling networks in PC

### 2.1 PC matrix-mediated drug resistance signaling networks

**FIGURE 1:**
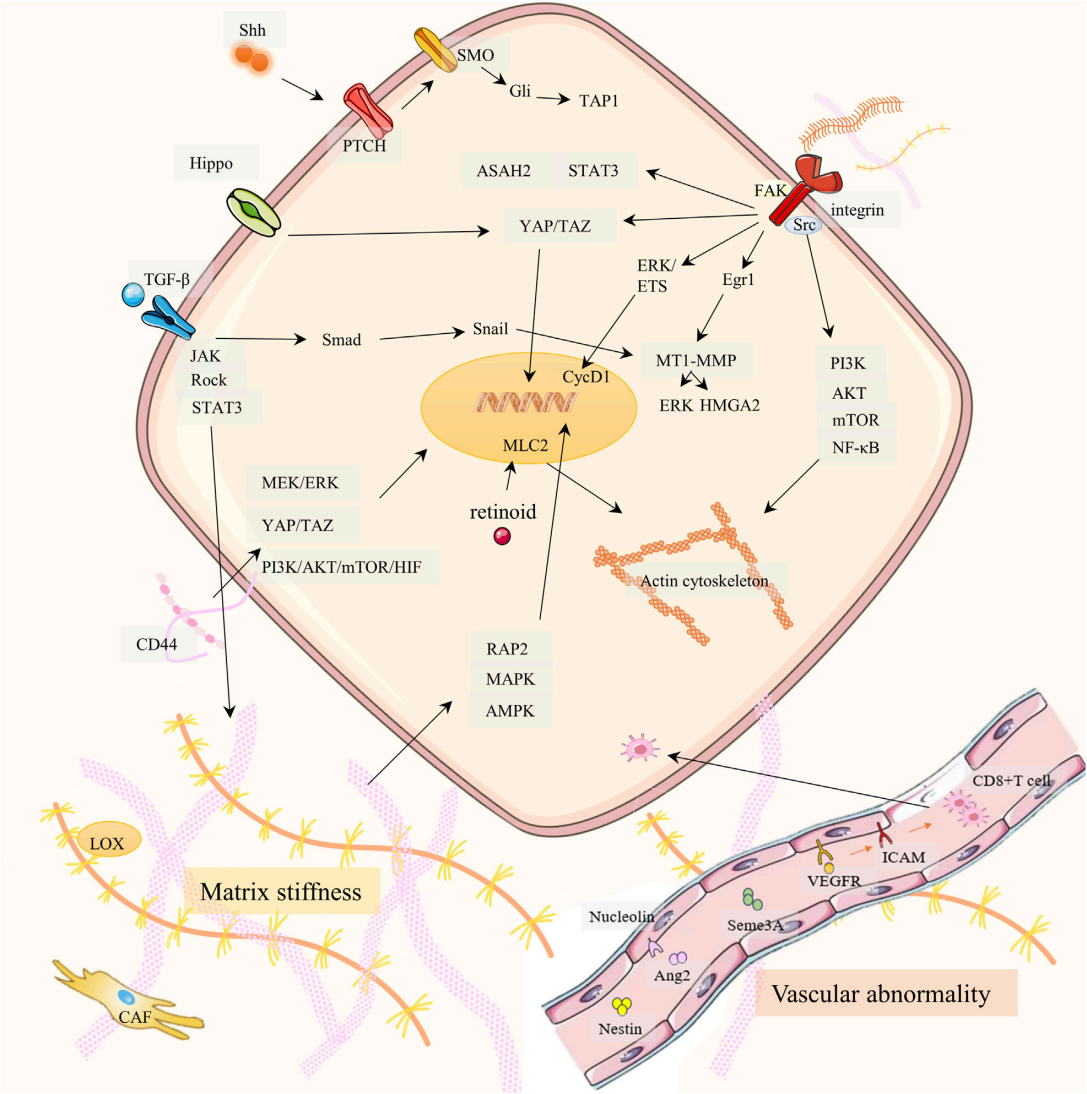
Drug resistance signaling networks targeting ECM. It is elaborated from three aspects: matrix stiffness (bottom left), vascular abnormality (bottom right), and ECM-receptor-mediated cancer cell migration (top).

The occurrence of PC not only depends on the instability and genetic mutation of its own genome but also needs the promotion of the external microenvironment. The TME in PC is characterized by significant fibril-matrix proliferation and lack of blood supply (Bear et al., 2020). The three main parts of the specific PC fibrous matrix are the ECM, the vasculature, and cancer-associated fibroblasts (CAFs). CAFs secrete ECM proteins, such as collagen and fibronectin, which, together with glycosaminoglycans such as hyaluronic acid (HA), increase interstitial pressure, leading to vascular collapse and hypoxia (Hosein et al., 2020). This process is crucial for the growth, migration, epithelial-mesenchymal transition (EMT), and treatment resistance of PC cells (Sherman and Beatty, 2023). In recent years, targeted matrix therapy has garnered significant attention, but has little effect (Polani et al., 2021). This may be related to stromal sclerosis, vascular abnormalities, and ECM-receptor interactions (Figure 1).

#### 2.1.1 Matrix stiffness

In most tumors, the stiffness is greater than that of normal tissues, and this stiffness is primarily determined by the rigidity of the ECM. The composition and proportion of the ECM largely dictate the stiffness of the matrix. Excessive collagen and hyaluronic acid (HA) are the main causes of ECM hardening (Giussani et al., 2015; Insua-Rodriguez and Oskarsson, 2016). A hard ECM can hinder drug delivery and lead to PC therapy resistance. PC has an overexpression of the extracellular enzyme lysyl oxidase (LOX), which increases the cross-linking of collagen fibers and hardens the ECM (Weniger et al., 2018; Kai et al., 2019; Kim et al., 2021). Cancer cells synthesize more collagen and HA and produce enzymes that promote ECM production, increasing ECM stiffness. HA receptor CD44 can activate PI3K-AKT, MEK-ERK, and RhoA pathways, and enhance PC therapy resistance (Tavianatou et al., 2019; Caon et al., 2020; Huang J. et al., 2021). TGF-β and CAF are also indirectly involved in stromal sclerosis. CAF promotes cancer cell proliferation by promoting ECM hardening and secreting cytokines. Both cancer cells and TAMs secrete TGF-β, which promotes fibroblasts to become CAFs. Therefore, a positive CAF-ECM-cancer cell cycle is formed (Huang J. et al., 2021). However, the effect of TGF-β on matrix stiffness is controversial in PC. Laklai H et al. discovered that damage to TGF-β signaling can promote JAK-ROCK-STAT3 signaling and increase matrix stiffness in PC (Laklai et al., 2016). In addition, increased ECM matrix stiffness can promote PC drug resistance, EMT, and metastasis through the RAP2-MAPK-AMPK pathway (Xu et al., 2024). Retinoids downregulate myosin-regulated light chain 2 (MLC-2) expression through RARβ/RXR, thereby reducing actomyosin activity and alleviating stromal sclerosis (Zhang et al., 2021).

#### 2.1.2 Vascular abnormality

Vascular abnormalities of PC are reflected in poor perfusion, hypoxia, leakage, and low pericellular coverage in the vasculature, which is not conducive to drug delivery (Li S. et al., 2019; Zhong et al., 2020; Huang et al., 2024). Studies by Groenewegen G et al. have shown that blocking the vascular endothelial growth factor (VEGF) -VEGFR2 axis through leukocyte adhesion molecules ICAM and VCAM can increase CD8^+^T cells and drug transport to tumors, improving hypoxia and drug resistance (Shrimali et al., 2010; Huang et al., 2012; Esmaeili et al., 2021). Maione et al. discovered that Sema3A functions as an endogenous and potent antiangiogenic agent that promotes vasculature normalization in a multistep carcinogenic PC mouse model (Maione et al., 2009). Nucleolin is a marker of angiogenesis in PC. Inhibition of Nucleolin can significantly reduce the expression of angiopoietin-2 (Ang-2) in endothelial cells, induce vascular normalization, improve gemcitabine delivery, and reduce drug resistance (Christian et al., 2003; Gilles et al., 2016). Nestin can promote vascular endothelial cell proliferation, and targeting Nestin can inhibit angiogenesis in a PC mouse model (Yamahatsu et al., 2012).

#### 2.1.3 ECM-receptor

The cytoskeleton, integrin, and other associated receptors play a major role in mediating adhesion between cancer cells and ECM components. If cell adhesion is disrupted, PC cells will migrate (Kanchanawong and Calderwood, 2023). Cancer metastasis and drug resistance generation share multiple signaling pathways (including integrins). Cancer cells of carcinoma *in situ* need to migrate into blood circulation and colonize a new environment. This process triggers a series of stress and repair responses that not only help the cells survive in their new environment but also help them become more resistant to treatment (Weiss et al., 2022). In addition, critical signaling pathways (e.g., PI3K-AKT, etc.) associated with cancer cell invasion also mediate drug resistance (Tufail et al., 2024). We summarize how ECM components (e.g., collagen, fibronectin, and laminin) interacting with receptors trigger downstream signaling pathways (including cell migration), which in turn affect drug resistance.

##### 2.1.3.1 Integrin family

Integrins act as the most important cell adhesion transmembrane receptor, linking the ECM-cytoskeleton and mediating various biochemical and mechanical signals. Each integrin is composed of α and β subunits. Collagen, fibronectin, and laminin in ECM can bind to integrin β1, which affects cell adhesion and drug resistance (Musiime et al., 2021; Kanchanawong and Calderwood, 2023; Pang et al., 2023). Integrin β1 (ITGB1) activates PI3K-p110β signaling, which in turn enhances gemcitabine resistance in PC (Yang et al., 2018). ITGB1 plays an important role in cell migration and may be responsible for the lumen formation of MEK inhibitors *in vitro* 3D PC models. Combined inhibition of ITGB1 and MEK increased the sensitivity of PC cells to MEK inhibitors (Brannon et al., 2020). The solute carrier SLC39A4 inhibits the expression of gemcitabine transporter ENT1 in PC cells by promoting integrin α3β1 signaling, thereby promoting drug resistance (Liu et al., 2020).

Some integrins that do not bind to ECM components can also function. For example, integrin α1 (ITGA1) is required for TGF-β/collagen-induced EMT and metastasis and cooperates with TGF-β to drive gemcitabine resistance (Gharibi et al., 2017). Integrin α2 may promote PC invasion and proliferation through phosphorylation of AKT, leading to gemcitabine resistance (Gregori et al., 2023). Integrin α5 is a ligand for the adhesion molecule L1CAM (CD171). L1CAM induces chemoresistance in PC, which may be related to IL-1β/NF-κB (Sebens Muerkoster et al., 2009; Kiefel et al., 2010). Integrin αvβ3/KRAS/NF-κB promotes serine/threonine kinase Tank-binding kinase 1 (TBK1) phosphorylation, which facilitates PC resistance, and is particularly highly resistant to tyrosine kinase receptor inhibitors (e.g., erlotinib) (Seguin et al., 2014; Cheng and Cashman, 2020). Integrin β5 overregulates the expression of sphingoid metabolizing enzyme ceramidase (ASAH2) through Src and STAT3 signal and reactivates pyroptosis to overcome PC chemoresistance (Su et al., 2023).

##### 2.1.3.2 Focal adhesion kinase (FAK)

FAK is a non-receptor tyrosine kinase activated by ECM receptors, including integrins (Macagno et al., 2014). Integrins activate the Src enzyme, and FAK binds to Src after autophosphorylation through the PI3K/AKT/mTOR pathway, causing a rise in NF-κB and cell migration. Through ERK/ETS signaling, activated FAK stimulates the overexpression of cyclin CycD1 and encourages the growth of cancer cells. In addition, FAK in the nucleus can also induce P53 degradation, helping cancer cells survive (Li B. Q. et al., 2019; Sciano et al., 2024). Several FAK inhibitors are in preclinical or clinical trials as potential chemotherapeutic agents for PDAC. Jiang et al. showed that prolonged FAK inhibition leads to downregulation of TGF-β, which enhances STAT3 signaling. This results in reduced collagen, reduced fibroblast numbers, and downregulation of TGF-β/SMAD signaling pathways in PC, resulting in resistance to FAK inhibition in PC (Jiang et al., 2020). Singh S et al. showed that CXCL12-CXCR4 also induces FAK activation, promotes migration, and facilitates PC gemcitabine resistance (Singh et al., 2010). In addition, FAK inhibitors overcame the obstacle of PC radiotherapy and achieved a good therapeutic effect when combined with an immune checkpoint inhibitor (ICI) (Lander et al., 2022).

##### 2.1.3.3 YES-associated protein (YAP) and PDZ-binding protein (TAZ)

Mechanical signals in the ECM activate YES-related proteins and PDZ-binding proteins, prompting their accumulation in the nucleus and triggering EMT. When intercellular adhesion is reduced, nucleus-activated YAP/TAZ binds to the transcription factor TEAD, expressing target proteins associated with cell migration, adhesion, stemness, and ECM remodeling (Kim et al., 2018; Eibl and Rozengurt, 2019; LeBlanc et al., 2021). These proteins upregulate gemcitabine resistance (Eibl and Rozengurt, 2019; Ma et al., 2024; Zhou et al., 2024). In addition, the Hippo/YAP1/c-Jun pathway can promote cancer cell stemness and iron metabolism in PC, promoting chemoresistance (Zhou T. et al., 2023). In addition, FAK can also activate YAP/TAZ (Ferrara et al., 2021).

##### 2.1.3.4 Matrix metalloproteinases (MMP)

MMP has collagen-binding domains that can move to collagen-susceptible sites (Van Doren, 2015). MMP not only mediates the degradation of ECM but also affects adhesion function (Ho et al., 2001; Niland et al., 2021). In previous studies, both the TGF-β/Smad3/Snail pathway and the collagen/ITGB1/Src/Egr1 signaling pathway upregulated membrane type 1 matrix metalloproteinase (MT1-MMP) (Shields et al., 2012). Munshi HG et al. first found that type I collagen increases ERK1/2 phosphorylation and high mobility group A2 (HMGA2) expression through MT1-MMP, and that HMGA2 is highly expressed in high-grade pancreatic tumors with lymph node metastasis, attenuating gemcitabine-induced checkpoint blockade (Dangi-Garimella et al., 2011; Dangi-Garimella et al., 2013). In addition, MMP-cleaved type I collagen remodeling activates the DDR1/NF-κB/p62/NRF2 signaling pathway to promote tumor metastasis regulates tumor growth and metabolism, and promotes PC growth (Su et al., 2022).

##### 2.1.3.5 Hedgehog signal

Instead of directly targeting specific components of the ECM, an alternative approach is to focus on specific signaling pathways for tumor stroma development (Ho et al., 2020). Hedgehog signaling is associated with drug resistance in PC. In the study of Khan MA et al., the co-culture of PC cells and pancreatic stellate cells (PSCs) promoted PC chemoresistance through Hedgehog and CXCR4 signaling (Khan et al., 2020). Sonic Hedgehog (Shh), a subtype of Hedgehog, activates the downstream factor Gli-1 by binding to Patched (PTCH), which then promotes PC chemoresistance with the participation of ABCB2 (Xu et al., 2013; Wang et al., 2019).

### 2.2 Cell-mediated drug resistance signaling networks

**FIGURE 2:**
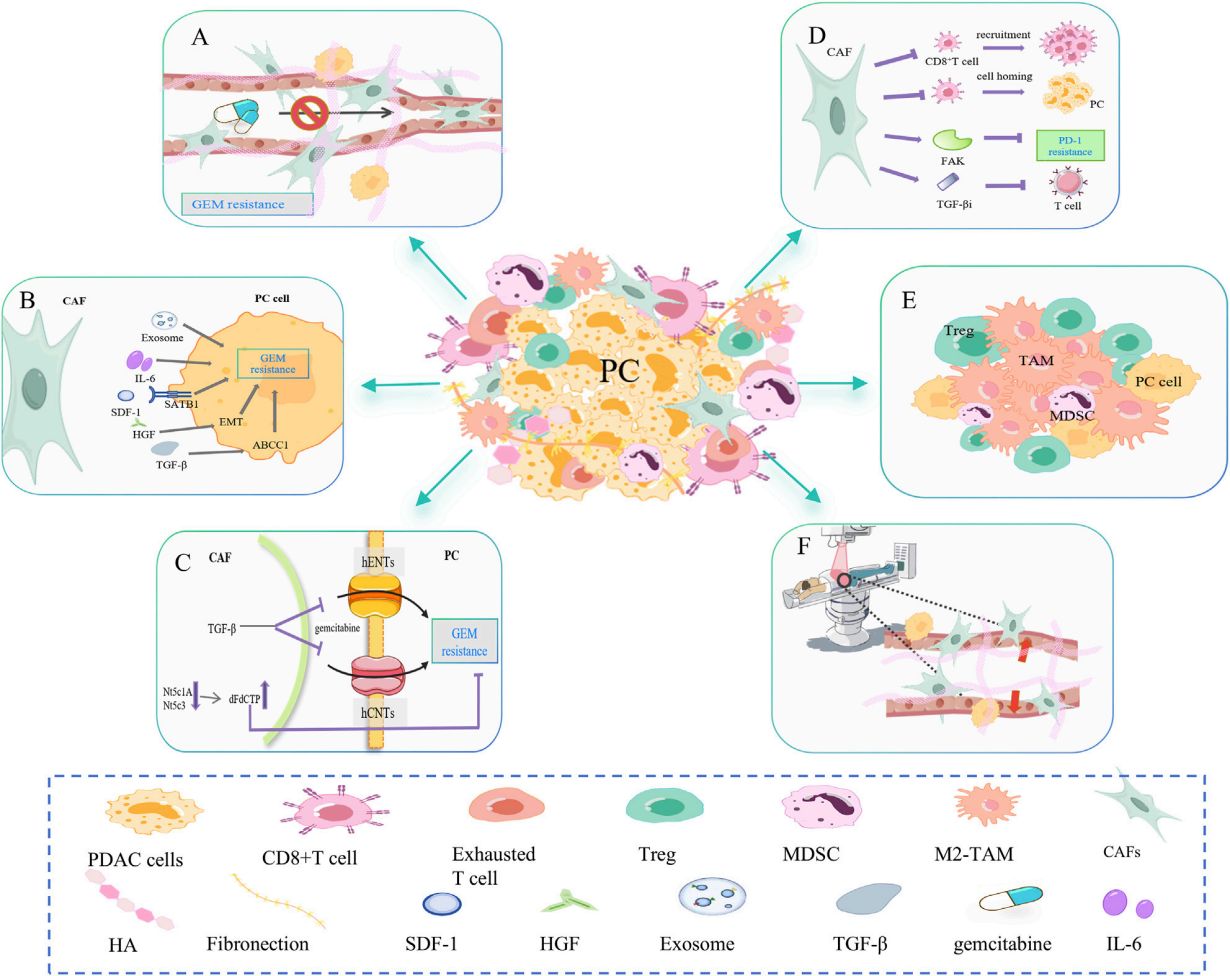
CAF-mediated resistance signaling networks to chemotherapy, immunotherapy, and radiotherapy. **(A)** CAF is involved in dense fibrosis in PC, forming a physical barrier. Excessive fibrosis leads to increased interstitial fluid pressure and vascular collapse, which affects the transport of gemcitabine in the blood. **(B)** CAF can secrete cytokines and exosomes such as IL-6, SDF-1, HGF, and TGF-β to promote gemcitabine resistance in PC cells. **(C)** CAF disrupts gemcitabine metabolism in cancer cells. On the one hand, CAF releases Nt5c1A and Nt5c3, resulting in high expression of gemcitabine in CAF, which indirectly reduces its concentration in PC cells. On the other hand, CAF inhibits the transporter proteins hENT1 and hCNT3, resulting in decreased gemcitabine uptake. **(D)** CAF inhibits the infiltration and function of CD8^+^ T cells. For example, CAF-released FAK promotes PD-1 inhibitor resistance. CAF-released TGFβi inhibits the activation and effector function of CD8^+^ T cells. **(E)** CAFs may increase the proportion of immunosuppressive cells in the PC microenvironment, such as M2-TAM, Treg, and MDSC. **(F)** CAFs mediate PC resistance to radiotherapy (RT) primarily by decreasing the oxygen concentration and altering the phenotype of the cancer cell.

**FIGURE 3:**
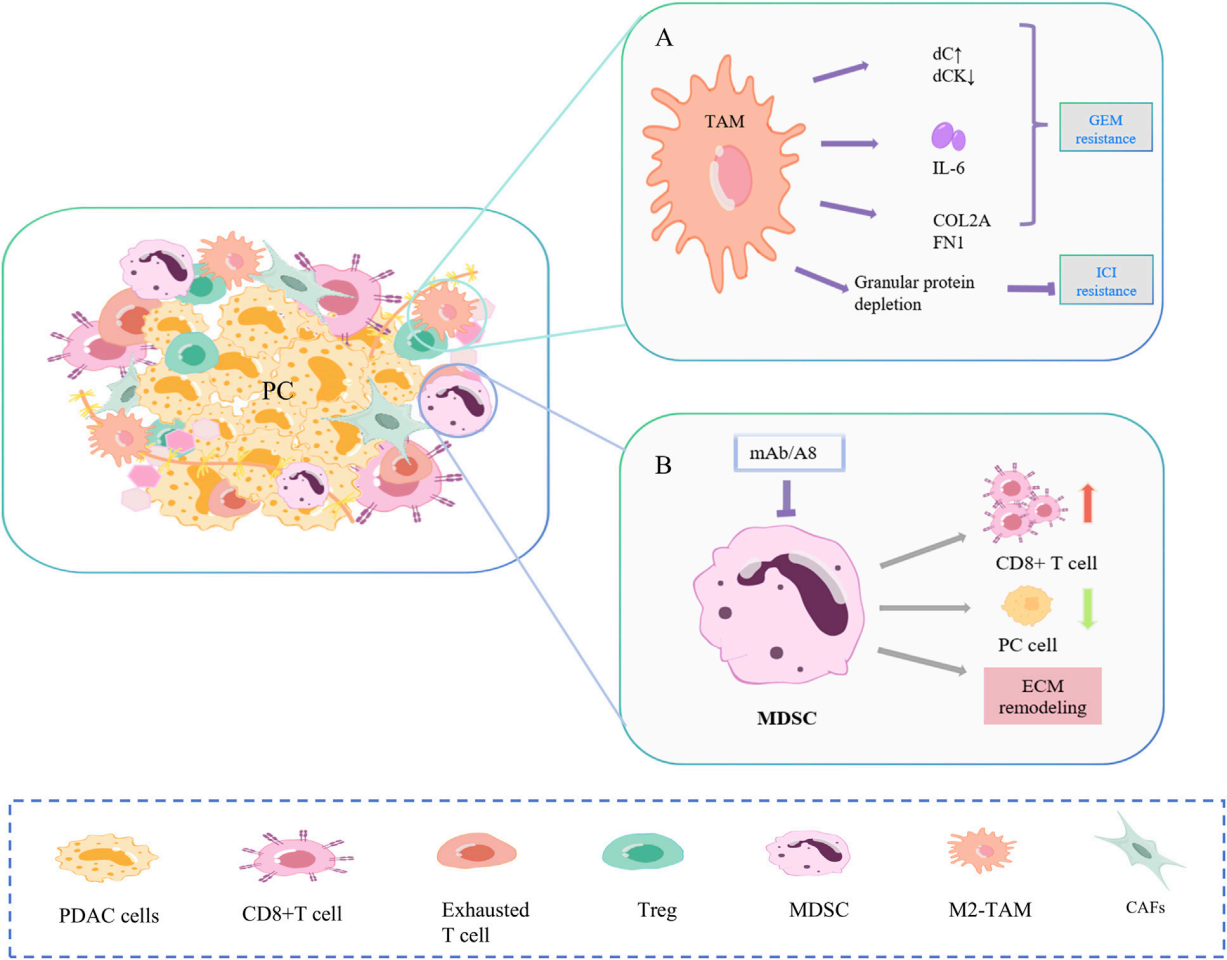
Mechanisms of drug resistance in TAM and MDSC. **(A)** TAMs promote gemcitabine resistance in PC cells primarily through secretion and polarization, and to ICI through reduced fibrosis and PD-1. **(B)** Targeted depletion of Gr-MDSC in PC increased activated CD8^+^ T cells, induced apoptosis in cancer cells, and also promoted stromal remodeling.

Resistance to PC therapy is largely attributed to the immunosuppressive TME, and we focused on immunosuppressive cells. CAFs accumulated significantly in TME. Several previous studies have demonstrated the pivotal function that CAFs and tumor cells perform in the initiation and advancement of malignancies (Rebelo et al., 2023). The interaction between immune cells and CAFs has received increased attention recently (Mao et al., 2021). By secreting various cytokines and exosomes, CAFs interact with tumor-associated macrophages (TAMs), dendritic cells (DCs), and other immune components to form immunosuppressive TME, causing immune escape of PC cells (Zhang et al., 2023a; Zhang et al., 2023b; Qin et al., 2024). We summarized the possible reasons for the suboptimal efficacy of contemporary therapies targeting these cellular components (Figures 2, 3).

#### 2.2.1 CAF

There have been conflicting results in recent clinical trials of CAF-targeted therapy for PC, and it seems that CAF has dual functions of pro- and anti-tumor in PC, possibly due to the fact that different subpopulations of CAF in the TME can be interconverted. This article only discusses the resistance signal networks of CAF-related chemotherapy, immunotherapy, and radiotherapy (Rebelo et al., 2023).

CAF changes drug resistance mainly by forming physical barriers, secretions and related signaling pathways, and drug metabolism. First, CAF is involved in the dense fibrosis of PC, forming a physical barrier (Figure 2A). Excessive fibrosis can lead to increased interstitial fluid pressure (IFP) and vascular collapse, affecting gemcitabine transport in the blood (Norton et al., 2020). The matrix depletion drug IPI-926 has been shown to improve gemcitabine resistance (Olive et al., 2009). As mentioned earlier, the consumption of HA also helps relieve matrix stiffness, allowing blood vessels to re-dilate and resume gemcitabine delivery (Jacobetz et al., 2013; DelGiorno et al., 2014). Secondly, CAF can secrete SDF-1, HGF, TGF-β, IL-6, and so on to promote the resistance of PC cells to gemcitabine (Figure 2B) (Neumann et al., 2018). SDF-1 is a specific C-C chemokine produced by CAF, which can upregulate special AT-rich sequence-binding protein 1 (SATB-1) through CXCR4, promoting PC progression and gemcitabine resistance (Wei et al., 2018). Hepatocyte growth factor (HGF) secreted by CAF promotes EMT, leading to intrinsic gemcitabine resistance (Feldmann et al., 2021). CAF secretes TGF-β1, which in turn activates the SMAD2/3-ATF4 axis/ABCC1 pathway. ABCC1 is a gemcitabine transporter that reduces the accumulation of gemcitabine in cancer cells and undermines the efficacy of gemcitabine (Robey et al., 2018; Wei et al., 2021). CAF can also release exosomes containing snail RNA and microRNA, promoting gemcitabine resistance (Richards et al., 2017; Richards et al., 2022). Finally, CAF disrupts gemcitabine metabolism in PC cells (Figure 2C). On one side, CAF will release low levels of hydrolytic cytosolic 5′-nucleotidases (Nt5c1A, Nt5c3), resulting in high expression of gemcitabine and its activated metabolite dFdCTP in CAF, indirectly reducing its concentration in PC cells (Hessmann et al., 2018). On the other side, PSCs are a significant source of CAFs. TGF-β-treated PSC overexpressed cysteine-rich angiogenic inducer 61 (CYR61), inhibited gemcitabine transporters hENT1 and hCNT3 and resulted in decreased gemcitabine uptake (Paproski et al., 2013; Hesler et al., 2016). In addition, CAF releases deoxycytidine, which rivals gemcitabine for phosphorylating deoxycytidine kinase (dCK) (Dalin et al., 2019).

CAF primarily inhibits CD8^+^T cell infiltration and activity (Figure 2D) and increases immunosuppressive cells (Figure 2E), which leads to PC immunotherapy resistance. First, CAF inhibits the recruitment and infiltration of CD8^+^T cells (Liu T. et al., 2019). Stiff ECM prevents CD8^+^T cells from homing to tumor tissue and restricts T cell movement (Hartmann et al., 2014). Targeted inhibition of FAK in ECM made previously unresponsive PC mice responsive to T-cell therapy and ICI (Jiang et al., 2016). Secondly, CAF inhibits the activation and effector function of CD8^+^T cells. The production of TGFβi by PDGFRα^+^ CAFs directly interacts with CD61 on the surface of CD8^+^T cells, thereby inhibiting T cell receptor signaling pathways. TGFβi depletion can significantly enhance the activation and function of CD8^+^T cells, increase the secretion of granzyme B, and inhibit tumor progression (Goehrig et al., 2019). In the study of Turley SJ et al., depletion of LRRC15^+^ CAF can improve anti-PD-L1 reactivity and weaken the inhibition of CD8^+^ T cell function (Krishnamurty et al., 2022). Furthermore, it has been demonstrated that CAFs can cause immune evasion by increasing the proportion of immunosuppressive cells in PC, such as Tregs, M2-phenotype macrophages, and metallogenic suppressor cells (MDSCs) (von Ahrens et al., 2017).

CAF mediates PC resistance to radiotherapy (RT) mainly by reducing oxygen concentration and changing cancer cell phenotype (Figure 2F). CAF produces excess ECM proteins, causing matrix stiffness and pressure on blood vessels resulting in hypoxia. Improving hypoxia can sensitize PC cells to ionizing radiation (Gray et al., 1953). In addition, cytokines secreted by CAF can alter the TME and may promote the transformation of PC cells to a radiation-resistant phenotype (Barker et al., 2015). During irradiation, the gene expression and phenotype of CAF are also altered, which in turn alters the ECM composition. This process promotes tumor angiogenesis, remodels immunosuppressive TME, and gradually makes pancreatic cancer cells resistant to radiotherapy (Li et al., 2016; Grinde et al., 2017; Nicolas et al., 2022; Guo et al., 2023; Zhang Y. et al., 2023).

#### 2.2.2 TAM

TAMs promote gemcitabine resistance of PC cells mainly through secretions and polarization (Figure 3A) (Hu et al., 2023; Zhou J. et al., 2023; Liu J. et al., 2024). Alternately activated macrophages, similar to CAF, release a series of pyrimidine nucleosides. Macrophages can produce more deoxycytidine (dC) and less dC kinase (dCK), resulting in chemoresistance (Halbrook et al., 2019; Zhang J. et al., 2023). TAM also secretes cytokines. IL-6β is low expressed in PC cells but highly expressed in TAMs and specifically mediates IL-6β-dependent gemcitabine resistance in TAMs (Spek et al., 2022). In the study of Zhou et al., TGFβ/TGFBI/Fn1 promoted the polarization of macrophages into TAM, increased the expression of ECM proteins COL2A and FN1, and promoted PC growth and gemcitabine resistance (Quaranta et al., 2018). In addition, the depletion of macrophage-derived granular protein reduced the formation of PC fibrosis and blocked the expression of PD-1, resulting in ICI resistance (Quaranta et al., 2018).

#### 2.2.3 MDSC

Two distinct subpopulations of MDSC, granulocytes (Gr-MDSC) and monocytes (Mo-MDSC), are involved in PC progression (Figure 3B). In Hingorani SR et al., targeted Gr-MDSC depletion activated CD8^+^T cells, triggered the apoptosis of PC epithelial cells, and also promoted stromal remodeling. In a study by Hingorani SR et al., targeted depletion of Gr-MDSC increased activated CD8 T cells, induced apoptosis in pancreatic cancer epithelial cells, and also promoted stromal remodeling. Although PC has low immunogenicity, Gr-MDSC depletion still triggers the adaptive immunity of PC (Stromnes et al., 2014).

### 2.3 Energy metabolism-mediated drug resistance signaling networks

Like other cancer cells, pancreatic cancer cells need to survive and proliferate in an environment where they lack oxygen and nutrients and are attacked by immune cells. This represents an enormous physical, oxidative, and inflammatory stress on PC cells. PC cells require metabolic reprogramming. On the one hand, expanding access to nutrients (macroautophagy and macropinocytosis (MP)). On the other hand, the “fuel” (glucose, amino acids, lipids) is fully utilized (Halbrook and Lyssiotis, 2017; Biancur and Kimmelman, 2018; Qin et al., 2020). PC cells have adapted metabolism to promote cell growth, while at the same time promoting chemoresistance.

#### 2.3.1 Energy acquisition

Macroautophagy is a highly conserved catabolic process, and autophagic flux increases in response to cellular stress (nutrient deprivation, hypoxia, and chemotherapy). Autophagy can have both pro- and anti-tumorigenic properties, and its role in tumorigenesis requires specific analysis (Gillson et al., 2022). Available evidence suggests that autophagy is increased in PC cells and promotes drug resistance (Liu Z. D. et al., 2024). Inhibition of autophagy in PC cells leads to impaired mitochondrial function, decreased oxidative phosphorylation (OXPHOS), and subsequently decreased ATP levels, reducing cancer cell survival (Yang et al., 2011; Yang et al., 2014). Single autophagy inhibitors are ineffective in patients with chemotherapy-resistant PC and can be combined with gemcitabine, MEK/ERK inhibitors, and radiation therapy (Wolpin et al., 2014; Bryant et al., 2019; Chen et al., 2021; Yazal et al., 2022). Autophagy also removes major histocompatibility class I (MHC-I) from the cell surface to impair recognition by the antitumor immune system and promote immune escape from PC cells, which is associated with ICI therapy resistance. Inhibition of autophagy restores surface levels of MHC-I, improves antigen presentation, and enhances ICB action (Yamamoto et al., 2020).

Unlike autophagy, which consumes the cell itself, MP internalizes extracellular substances. Mutant KRAS (mKRAS) upregulates syndecan-1 (SDC1) on the cell surface, which in turn promotes MP (Yao et al., 2019; Hobbs et al., 2020). MP plays a dual role in the treatment of PC. On the one hand, MP inhibitors can combine with autophagy inhibitors to induce a decrease in tumor metabolism and treat PC (Su et al., 2021). Since lysosomal activity is essential for both autophagy and macropinocytosis, lysosomal inhibitors and mKRAS signaling inhibitors show synergistic antitumor activity (Bryant et al., 2019; Kinsey et al., 2019). On the other hand, albumin can enter mKRAS-driven cancer cells in large quantities via MP, reinforcing the role of drug delivery carriers (Wang et al., 2018). For example, Liu et al. found that mKRAS caused enhanced MP and decreased expression of neonatal Fc receptor (FcRn), sensitizing PC cells to albumin-coupled adriamycin (Liu H. et al., 2019).

#### 2.3.2 Energy utilization

Specific metabolic abnormalities in PC cells are strongly associated with chemoresistance, particularly the increased use of glucose and the metabolism of the amino acid glutamine to power cancer cells. Many oncogenic activations in PC (e.g., KRAS, TP53, and MYC) have glycolytic activity-promoting effects. Shukla et al. found that hypoxic TME activates HIF-1α, which mediates an increase in glycolytic flux, glucose addiction in cancer cells, a corresponding increase in pyrimidine biosynthesis, and an increase in deoxycytidine triphosphate (dCTP), and that the elevated level of dCTP reduces the effective level of gemcitabine through molecular competition. The increase in dCTP levels decreases the effective level of gemcitabine through molecular competition, which ultimately leads to the development of gemcitabine resistance in PC cells (Shukla et al., 2017). Targeting key enzymes and molecules in metabolism (mTOR, hexokinase, LDH-A, E2F1, etc.) can reduce PC chemoresistance (Boudreau et al., 2016; Feng et al., 2018; Fan et al., 2019). Combining metabolic inhibitors with standard therapies produces synergistic effects that enhance cancer treatment. Although most inhibitors are still in the preclinical stage, glycolytic enzyme inhibitors represent a promising anticancer therapy (Grasso et al., 2017).

Another pathway for generating energy, OXPHOS, is significantly increased in cancer stem cells (CSCs). Targeting OXPHOS eliminates CSCs and attenuates cancer drug resistance (Zhao et al., 2022). Patients with high OXPHOS have a lower overall survival and a poorer prognosis than patients with low OXPHOS. High OXPHOS status was positively correlated with mitochondrial complex I abundance, and the combination of gemcitabine and phenylbiguanide (targeting mitochondrial complex I) significantly improved the efficacy of gemcitabine, probably because highly active mitochondrial respiration better maintains resistance to gemcitabine-induced stress, a pro-survival process that is reduced by OXPHOS inhibition (Masoud et al., 2020).

PC cells use glutamine (Gln) to support proliferation and redox homeostasis. Recently, Kimmelman et al. found that the Gln antagonist DON significantly impaired the metabolism of PC cells and inhibited tumor growth and that the combination of a Gln inhibitor and trametinib had better therapeutic effects (Encarnación-Rosado et al., 2024). In addition, Gln addiction is also important for controlling ROS generation and activating mTOR, both of which can lead to chemoresistance (Chen et al., 2017; Feng et al., 2018).

In lipid metabolism, fatty acid synthase (FASN) expression is upregulated, which is also critical for gemcitabine resistance. Overexpression of FASN in PC cells upregulates Pyruvate Kinase M2 (PKM2) expression, promoting glycolysis and gemcitabine resistance (Tian et al., 2018). PKM2 also plays a non-metabolic role in chemoresistance by inhibiting gemcitabine-induced TP53 signaling and subsequent apoptosis (Kim et al., 2015). Orlistat, a FASN inhibitor, induces ER stress and increases gemcitabine sensitivity in an *in situ* PC mouse model (Tadros et al., 2017).

## 3 Drug resistance signaling networks in PC therapeutic strategies

### 3.1 Gene-targeted therapy

**FIGURE 4:**
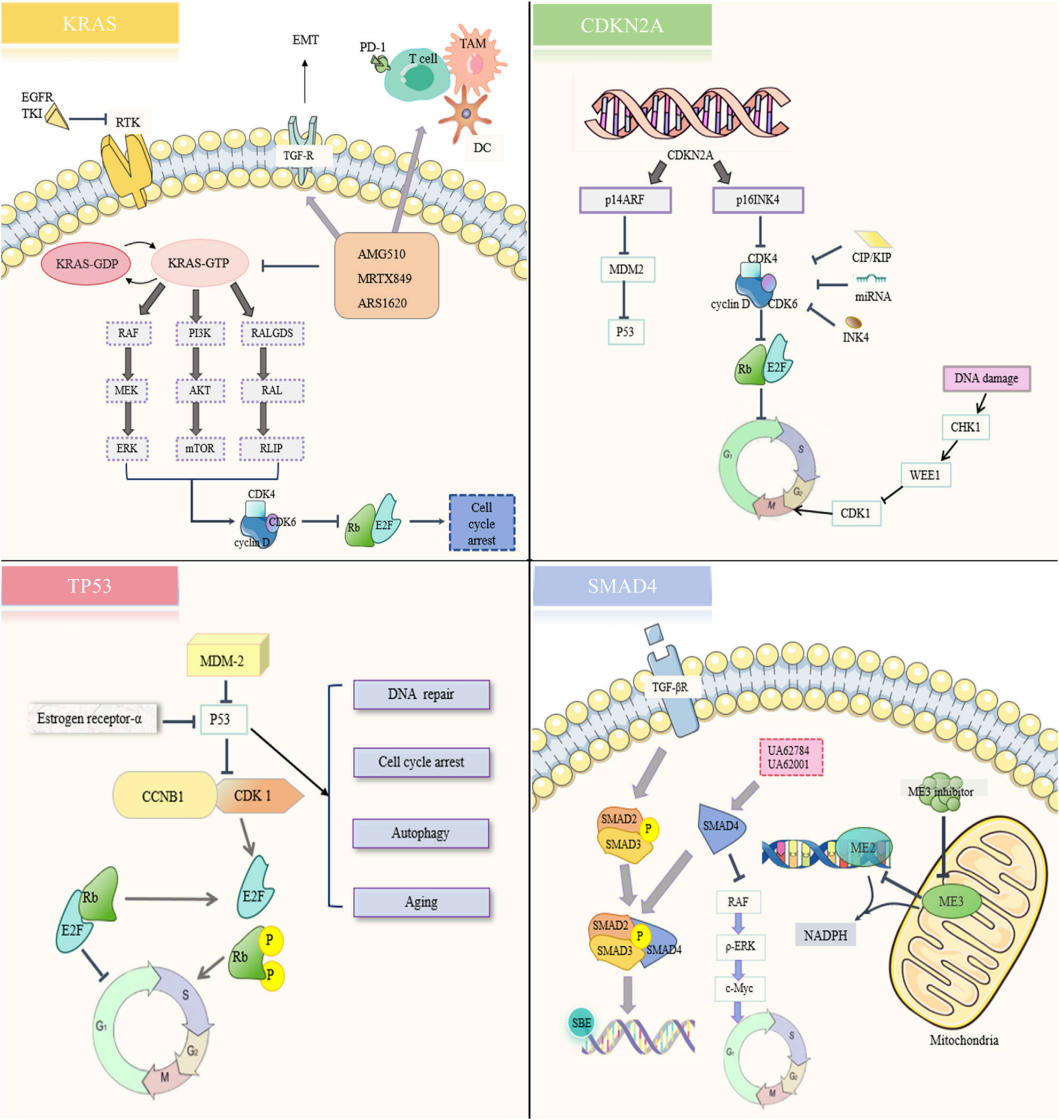
Resistance signaling network of immunotherapy targeting four mutated genes.

Therapy resistance in PC is also closely related to its unique epigenetic mechanism. Early-stage PC is primarily driven by mutations in four key genes: KRAS, CDKN2A, TP53, and SMAD4. The sequence of PC mutations is derived from studies of PC precancerous lesions, also known as PanIN. KRAS mutations occur in PanIN-1A and PanIN-1B, CDKN2A mutations occur in PanIN-2, and TP53 and SMAD4 mutations occur in PanIN-3 (Wang et al., 2021). We summarized the resistance signaling networks downstream of the four genes (Figure 4) (Qian et al., 2020).

#### 3.1.1 KRAS

KRAS is an oncogene that is a member of the RAS protein family and codes for guanosine triphosphate (GTPase). The RAS protein family consists of KRAS, NRAS, and HRAS. Under normal circumstances, KRAS cycles between activated and inactivated states. When KRAS binds to GTP via guanine nucleotide exchange factor (GEF), KRAS appears to be activated. When KRAS is bound to GDP by GTPase activating protein (GAP), KRAS is inactivated. mKRAS can lead to decreased GAP activity, resulting in excessive accumulation of activated KRAS, excessive activation of downstream pathways, and promoting tumor progression (Punekar et al., 2022). mKRAS, for example, may cause the RAF/MEK/ERK, PI3K/AKT/mTOR, RALGDS/RAL/RLIP pathway activation, and promote PC cell proliferation and invasion (Khan et al., 2021). In addition, mKRAS can also promote immunosuppressive TME formation (Bear et al., 2020).

New approaches to targeting mKRAS are rapidly evolving, and KRAS is gradually being transformed from an undruggable to a druggable target (Ostrem and Shokat, 2016). Targeted inhibitors against KRAS G12C mutations (Sotorasib and Adagrasib) have made significant progress in non-small cell lung cancer (Huang L. et al., 2021; Koga et al., 2021). However, the most common KRAS mutations in PDAC are G12D (44%), G12V (34%), and G12R (20%), which cannot be treated by small molecule inhibitors targeting G12C (Stickler et al., 2024). MRTX1133, a small molecule inhibitor targeting G12D, is currently under clinical investigation (Wang et al., 2022; Wei et al., 2024). However, not all G12D-mutated cell lines are sensitive to MRTX133, which may be due to mutations at other points near the switch II pocket or alternative signaling activation (Singhal et al., 2024).

In addition to directly targeting KRAS G12D, other drugs that target KRAS and its upstream and downstream sighnals may have several resistance mechanisms as follows: (1) The binding process of KRAS and GTP is not successfully inhibited; (2) The downstream signal of mKRAS reduces the expression of MHC-I and increases the expression of immune checkpoint PD-L1 and CD47 by enhancing autophagy, thereby directly avoiding anti-tumor immunity (El-Jawhari et al., 2014); (3) Activation of KRAS upstream receptor tyrosine kinases (e.g., EGFR) promotes the production of more RAS proteins and drug resistance (Amodio et al., 2020; Akhave et al., 2021); (4) Constitutive AKT could bypass KRAS-induced apoptosis and continue to promote PC growth (Pettazzoni et al., 2015); (5) Cell cycle kinase CDK4/6 promotes cancer cell proliferation (Goodwin et al., 2023); (6) Low antigen specificity (T cells), weak effect of innate (TAMs) and adaptive (CAFs) immune cells, strong effect of immunosuppressive cells (antigen-presenting cells, MDSCs) (Bear et al., 2020).

#### 3.1.2 CDKN2A

CDKN2A, a cell cycle protein-dependent kinase inhibitor, is one of the most important oncogenes. Two proteins encoded by CDKN2A, p16INK4, and p14ARF, control the cell cycle through CDK4/6 and MDM2 (murine double minute 2, a negative regulator of p53), respectively. There are currently no drugs targeting CDKN2A, but there is palbociclib, a small molecule inhibitor targeting CDK4/6. Palbociclib enhanced the therapeutic effect of gemcitabine and inhibited PC cell invasiveness by inducing apoptosis and cell cycle arrest and destroying the surrounding ECM tissue (Chou et al., 2018).

The resistance mechanism of CDK4/6 inhibitor may include: (1) The deficiency of cell cycle specific protein RB; (2) RB-E2F complex amplification; (3) INK4 family members overexpress competitive binding CDK4/6; (4) Loss of CDK interaction protein/kinase inhibitor protein (CIP/KIP) family expression; (5) Overexpression of WEE1 G2 checkpoint kinase (WEE1); (6) miRNAs (miR-138, miR-506, miR-6883-5p) directly target CDK4/6 and antagonize CDK4/6 inhibitors; (7) Other cell cycle non-specific pathways (Huang J. et al., 2022).

#### 3.1.3 TP53

The TP53 gene is the most frequently mutated in all cancers and encodes the P53 transcription factor (Junttila and Evan, 2009). P53 is activated in response to a variety of cellular stress signals such as hypoxia and DNA damage. After the activation of P53, it plays the functions of DNA repair, cell cycle arrest, autophagy, and aging through downstream signals. MDM2 acts as a negative regulator of p53 and regulates its functions. However, P53 mutations have been observed in most PC samples (Ryan et al., 2014). P53 mutation (mut-P53) promotes the transformation of precancerous lesions to PC (Morton et al., 2010). At present, there are two main therapies for P53: deleting the mut-P53 protein and restoring wild-type P53 activity (Parrales and Iwakuma, 2015).

The mechanism of drug resistance of TP53 therapy may include: (1) MDM2 overexpression; (2) Loss of DNA mismatch repair (Wang et al., 2001; Hientz et al., 2017); (3) Overexpression of mut-P53 target genes CDK1 and CCNB1 (Dhayat et al., 2015; Fiorini et al., 2015); (4) Estrogen receptor-α overexpression (Liu et al., 2006).

#### 3.1.4 SMAD4

SMAD4 is a tumor suppressor gene involved in TGF-β signaling. TGF-β forms a complex with SMAD2/SMAD3/SMAD4 and binds to other transcription factors and SMAD-binding elements to function in DNA repair, cell cycle arrest, and inhibition of proliferation (Derynck et al., 2001; Shi and Massague, 2003). Although there are currently anti-cancer drugs that target SMAD4-deficient cells, more research is needed to verify them (Wang et al., 2006; Wang et al., 2009).

Targeted SMAD4 therapy resistance mechanism may be: (1) Overexpression of mitochondrial malic enzymes 3 (ME3) (Dey et al., 2017; Sheth et al., 2023); (2) Excessive activation of RAF/ERK/c-Myc signaling pathway; (3) Increased mitochondrial autophagic flux driven by MAPK/ERK signaling (Ezrova et al., 2021).

### 3.2 Immune checkpoint inhibitor therapy

**FIGURE 5:**
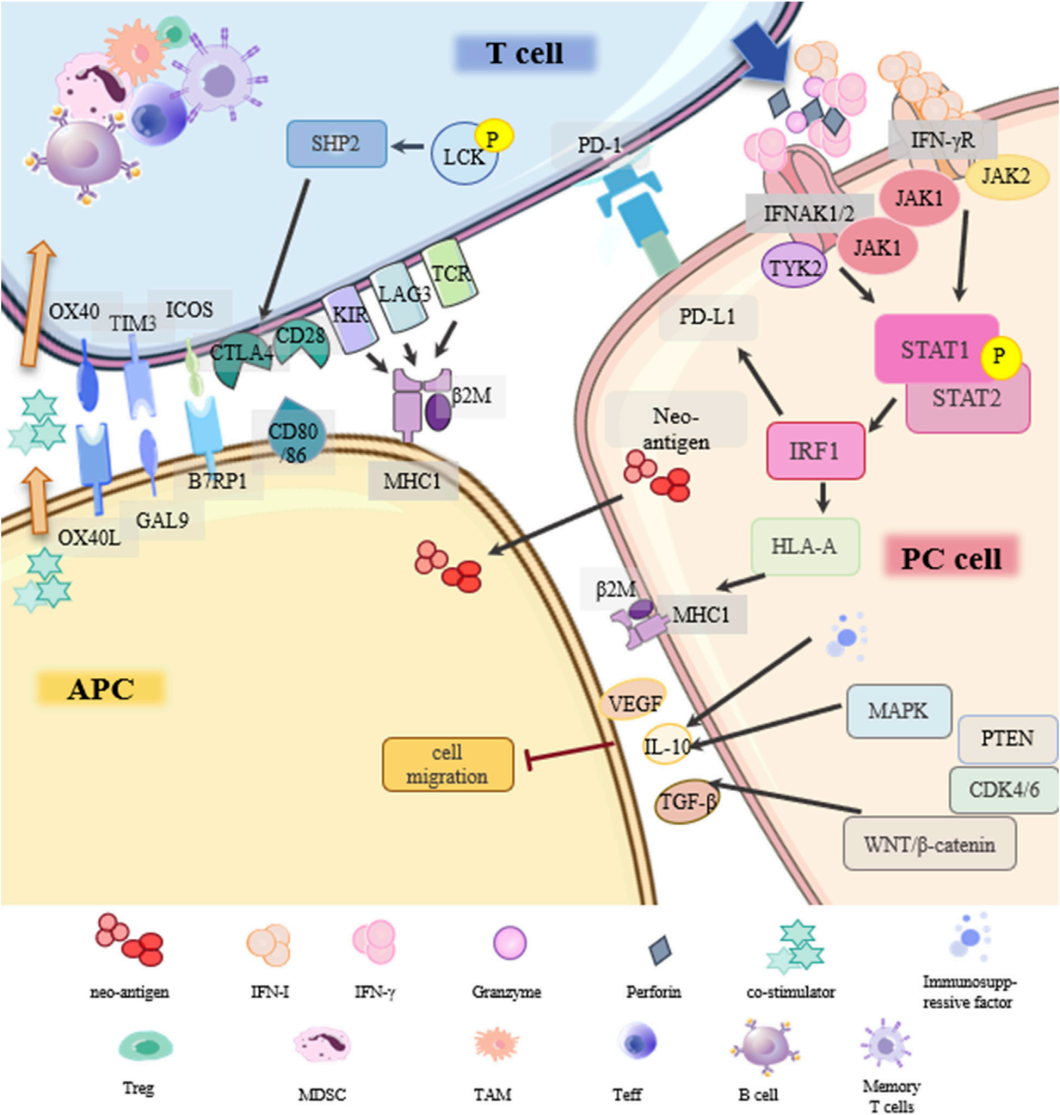
Drug resistance signaling network of immunosuppressive therapy.

ICIs play a key role in cancer immunotherapy and are often used in combination therapy for PC (Tang et al., 2021). This is because PC, as a “cold” tumor, has strong drug resistance and low immunogenicity, and a single ICI has almost no effect on PC (Micevic et al., 2023). Identifying signal networks for ICI resistance can help overcome this critical challenge (Figure 5).

Programmed cell death protein-1 (PD-1) is expressed in immune cells. Its ligand, PD-L1, is expressed in antigen-presenting cells (APCs) and tumor cells. The main resistance mechanisms of PD-1/PD-L1 inhibitors in PC include: (1) Disruption of PD-1/PD-L1 binding; (2) Low immunogenicity in PC; (3) Deficiency in antigen presentation; (4) Lack of suitable/effective neoantigens; (5) Other underlying factors.

Under normal circumstances, tumor cells release antigens and T cells respond by releasing interferon IFN-γ. IFN-γR on PC cells activates the JAK-STAT pathway, activates the transcription factor interferon regulatory factor 1 (IRF1), and triggers the production of PD-L1. If the PC cell is not responsive to IFN-γ, it will not express PD-L1, and the PD-1/PD-L1 inhibitor is ineffective (Ding et al., 2019). In addition, abnormal expression of MHC results in loss of immunogenicity, and PC cells cannot be completely cleared (Cabrera et al., 2003). IFN-γ signaling can enhance MHC-I antigen presentation by affecting the transcription of human leukocyte antigen HLA-A. TCR on the surface of T cells recognizes MHC on tumor cells and promotes the killing of PC cells. Tumor antigen presentation occurs mainly through the MHC-I pathway, defects in this pathway are more frequently observed than defects in MHC-II antigen presentation (Kalbasi and Ribas, 2020). LAG3 is a ligand for MHC-II and a common immune checkpoint receptor that reduces the effecter T cell activity and proliferation. Baleeiro et al. found that the MHC-II/LAG-3 pathway contributes to the killing of PC cells by T cells. This contradicts the role of LAG3 as an immune checkpoint in other cancers, which may be due to the fact that LAG3 can play different roles depending on the activation status of the T cells (Baleeiro et al., 2022). In addition, when cancers develop, they accumulate mutations that form some new autoantigen epitopes that are immunogenic and are called “neoantigens”. PC, though it has a longer time to accumulate mutations, produces fewer neoantigens (Luksza et al., 2022). Finally, the immunosuppressiveTME (immune cells and cytokines) unique to PC, changes in T cells (insufficient production, poor effect, impaired memory), and various classical cancer signaling pathways (TGFβ, WNT-β, CDK4/6, MAPK, PTEN) may be involved in the resistance of PD-1/PD-L1 inhibitors. Further verification is required (Jenkins et al., 2018; Tang et al., 2022).

CTLA4 is the first clinically targeted immune checkpoint receptor and is expressed only on T cells. Similarly, sensitivity to CTLA4 blockade is lacking due to insufficient antigenicity of PC tumors (Pardoll, 2012; Kalbasi and Ribas, 2020). Other emerging immune checkpoints such as TIGIT, GITR, LAG3, TIM3, and other drug-resistance signal networks in PC still need to be further explored (Borgeaud et al., 2023).

### 3.3 Cancer vaccines

#### 3.3.1 mRNA vaccine

Compared with DNA vaccines, mRNA vaccines can naturally target APCs and express a variety of powerful antigens without the need for transgenes to enter the nucleus and avoid potential toxic reactions. mRNA vaccine particle (MVP), after ingestion by DC, triggers the production of IFN-β and TNF-α via IFN gene stimulator factor (STING) and mitochondrial antiviral signaling (MAVS), respectively. T cells are then turned on to destroy antigen-specific PC cells (Chen et al., 2023). More recently, patients with surgically excised PC have been treated with an RNA-based tailored neoantigen vaccine (BNT122), immune checkpoint therapy (atezolizumab), and chemotherapy (mFOLFIRINOX) to produce a neoantigen-specific T cell response and postpone PC recurrence (Rojas et al., 2023). However, the application of mRNA vaccines is limited by instability, innate immunogenicity, and inefficiency of delivery *in vivo* (Miao et al., 2021).

#### 3.3.2 Peptide-based vaccine

Antigen epitope is the smallest immunogenic region of antigen, and peptide vaccines are developed based on antigen epitope. Peptide vaccines, after being presented with APC, activate the immune response and promote activated T cell proliferation and immune memory formation (Hu et al., 2021). In precancerous lesions of PC, such as PanIN and IPMN, previous research has found cancer-causing mutations in genes including GNAS and KRAS (Fischer and Wood, 2018). The KRAS-targeted peptide is the first polypeptide vaccine to undergo clinical trials. In addition, there are peptide- and protein-based vaccines targeted by telomerase, gastrin, survivin, vascular endothelial growth factor receptor (VEGFR), and HSP-peptide complexes (McCormick et al., 2016). At present, peptide vaccines are still limited by MHC and only activate monoclonal T cells, which may lead to a reduced anti-tumor immune response (Matsui et al., 2018).

#### 3.3.3 Whole-cell vaccine

Autologous whole-cell tumor vaccines are typically tumor cells that have been treated to remove tumorigenicity, retain their immunogenicity, and contain a full range of tumor antigens. However, it is still limited by low immunogenicity and high tumor heterogeneity. As one of the neoadjuvant treatments for PC, pancreatic tumor whole cell vaccine (GVAX) stimulates the secretion of GM-CSF and promotes DC activation. GVAX can increase the effectiveness of ICI by reprogramming the TME of PC and causing PD-L1 to be upregulated in tertiary lymphoid aggregates (Lutz et al., 2014; Soares et al., 2015; Wang et al., 2024). Algenpantucel-L can express natural immunogenic epitope α-galactose, promoting epitope spreading and immune response, eventually leading to hyperacute rejection and killing PC cells (McCormick et al., 2016; Hewitt et al., 2022).

#### 3.3.4 DC vaccine

As the most effective APC, DC vaccines are usually loaded with tumor-associated antigen (TAA) and reinfused into the patient. The DC vaccine’s effectiveness is still quite low, mainly because of insufficient tumor antigen expression and immunosuppressive TME. Tumor cells under-express TAA on the one hand and produce inhibitory factors to suppress DC differentiation and maturation on the other. In addition, immune checkpoints expressed on the DC surface (PD-1, PD-L1, and TIM-3) can also impair its function (Zhang X. et al., 2023). Recently, Zhang et al. found that DC vaccines expressing MUC1, MUC4, wilms tumor gene-1 (WT1), and KRAS antigens can enhance CTL response and prolong the median OS in PC patients (Zhang X. et al., 2023). It has recently been shown that the fusion vaccine, which combines autologous dendritic cells with primary tumor cells, activates and expands lymphocytes more successfully and has superior anticancer effects (Orr et al., 2023).

#### 3.3.5 Microbiome-based vaccine

Microbial-based vaccines use natural immunogenic viruses or bacteria as vectors that are engineered to express TAA. For example, CRS-207 (Live-attenuated *Listeria* monocytogenes expressing mesothelin) can be used in combination with GVAX and cyclophosphamide (Cy) to treat PC (Tsujikawa et al., 2020). Mesothelin is a tumor antigen that is highly expressed in PC. *Listeria* can induce IFNβ expression through an STING-dependent pathway (Tsujikawa et al., 2020). In addition, we first induced an initial T-cell response with a novel vaccinia virus vector expressing the tumor antigens CEA and MUC-1, as well as three co-stimulatory molecules, followed by an enhanced immune response with the same expression of a non-replicating fowlpox virus in PC patients. However microbial-based vaccines require complex engineering systems and careful manufacturing, which is not conducive to their personalized therapeutic applications.

#### 3.3.6 Exosome-based vaccine

Numerous tumor antigens are found in tumor-derived exosomes (TEXs), which can encourage DC binding and exosome absorption. TEXs have promising development potential and could represent the next-generation of cell-free vaccinations (Dai et al., 2024). Currently, the use of DC-derived exosome vaccines (DCexos) has not been able to stimulate T-cell responses and has only little clinical efficacy. This may be because all current clinical trials use monocyte-derived DC, which is not the optimal DC. Lack of adequate maturation/adjuvant signal is also a possible cause (Yao et al., 2021). In the study of Li et al., DCexos activated T-cell responses in PC mice and played an antitumor role (Xiao et al., 2017). It is worth noting that TEXs also include proteins and nucleic acids, which may cause autoimmune diseases by upsetting the immune system’s homeostasis after vaccination, raising concerns about the safety of precision therapy (Sun et al., 2020).

## 4 Clinical trials

We summarized existing clinical trials of various treatments for PC (Table 1). With every negative clinical trial, it is not hard to see that monotherapy is unlikely to succeed in PC, while combination therapies hold the most promise in the short term. Mitigating drug resistance can start with combining the key nodes of the signaling network and enhancing the combination of various therapies.

**TABLE 1:** Existing clinical trials of various treatments for PC.

Therapy	Study title	NCT number	Phase	Conditions
PC matrix-targeted therapy	Second-line Study of PEGPH20 and Pembro for HA High Metastatic PDAC	NCT03634332	Ⅱ	Pancreatic Ductal Adenocarcinoma; Pancreatic Cancer; Pancreatic Neoplasms
	Stereotactic Body Radiotherapy and Focal Adhesion Kinase Inhibitor in Advanced Pancreas Adenocarcinoma	NCT04331041	Ⅱ	Pancreas Cancer; Pancreas Adenocarcinoma
	Hedgehog Inhibitors for Metastatic Adenocarcinoma of the Pancreas	NCT01088815	Ⅱ	Metastatic Pancreatic Cancer
Cell-targeted therapy	Studying Fibroblast Activity in Patients With Localized Pancreatic Cancer Undergoing Surgery	NCT00900016	Unknown	Pancreatic Cancer
	Macrophages Effect on Chemoresistance	NCT01921699	Unknown	Pancreatic Cancer
Metabolism-targeted therapy	A Phase I/II/Pharmacodynamic Study of Hydroxychloroquine in Combination With Gemcitabine/Abraxane to Inhibit Autophagy in Pancreatic Cancer	NCT01506973	Ⅰ/Ⅱ	Pancreatic Cancer; Pancreas Adenocarcinoma
	Investigating Targetable Metabolic Pathways Sustaining Pancreatic Cancer	NCT05296421	Unknown	Pancreatic Cancer
Gene-targeted therapy	Selumetinib Sulfate in Treating Patients With Locally Advanced or Metastatic Pancreatic Cancer With KRAS G12R Mutations	NCT03040986	Ⅱ	Stage III Pancreatic Cancer; Stage IV Pancreatic Cancer
	Mutation of K-RAS, CDKN2A, SMAD4, and TP53 in Pancreatic Cancer: Role of Liquid Biopsy in Preoperative Diagnosis	NCT03524677	Unknown	Pancreatic Cancer
Immune checkpoint inhibitors	SBRT and Anti-programmed Cell Death Protein 1 (Anti-PD-1) in Late Stage or Recurrent Pancreatic Cancer Patients	NCT03716596	Ⅰ	Pancreatic Cancer
	Immune Checkpoint Inhibition in Combination With Radiation Therapy in Pancreatic Cancer or Biliary Tract Cancer Patients	NCT02866383	Ⅱ	Metastatic Pancreatic Cancer; Metastatic Biliary Tract Cancer
Cancer vaccines	Pooled Mutant KRAS-Targeted Long Peptide Vaccine Combined With Nivolumab and Ipilimumab for Patients With Resected Mismatch Repair Protein (MMR-p) Colorectal and Pancreatic Cancer	NCT04117087	Ⅰ	Colorectal Cancer; Pancreatic Cancer
	GVAX Pancreas Vaccine (With CY) in Combination With Nivolumab and SBRT for Patients With Borderline Resectable Pancreatic Cancer	NCT03161379	Ⅱ	Pancreatic Cancer
CAR-T	CD276-targeted Chimeric Antigen Receptor T Cells in Treatment With Advanced Pancreatic Cancer	NCT05143151	Ⅰ/Ⅱ	Advanced Pancreatic Carcinoma
	Mesothelin-targeted CAR-T Cells as a Neo-adjuvant Treatment in Patients With Resectable Pancreatic Cancers: a Feasibility Study	NCT06054308	Unknown	Pancreatic Cancer

## 5 Conclusion

PC is one of the most immune-resistant tumors, a characteristic determined by its unique immunosuppressive matrix and genomic landscape. Stiff matrix, abnormal vascularization, ECM-receptor-mediated signaling, and immunosuppressive cellular components are the main causes of resistance to targeted stroma therapy. In addition, PC is characterized by genomic instability. In pancreatic intraepithelial neoplasia, four common mutated genes play a key driving role in promoting the development of PanIN to PC. In addition, other carcinogenic drivers disrupt T-cell immunity in the early stages of tumor initiation, which partly explains the failure of ICI treatment. Cancer vaccines still need to be used in combination with other medications to overcome immunosuppression because of the lack of effector T cells and low PD-L1 levels.

Furthermore, other treatments for PC also have certain limitations. CAR-T therapy uses genetic engineering to transfer an engineered T cell receptor or chimeric antigen receptor into T cells. These T cells are then transfused back to destroy tumor cells that express specific tumor antigens. The reason for treatment limitation may be: (1) CAR-T cell exhaustion; (2) Antigen escape; (3) Physical barrier formed by PC matrix; 4) Immunosuppressive TME (Akce et al., 2018; Ma et al., 2019; Hagel et al., 2023). Senescence induction therapy is also getting more attention. In addition to inhibiting PC cell proliferation, this therapy improves drug delivery and efficacy by restoring blood supply through pro-angiogenic agents. However, because senescent cell escape and senescent cell accumulation can promote PC, senescence induction therapy still needs to be further explored (Chambers et al., 2021; Chibaya et al., 2022; Bordon, 2023; Chibaya et al., 2023). CD40 agonists can activate T cell immunity, activate macrophages and DCs, and destroy the PC tumor matrix. However, due to the lack of tumor-infiltrating lymphocytes in the TME, T cell function is impaired and its application is limited (Vonderheide et al., 2013; Vonderheide, 2018; Djureinovic et al., 2021).

In summary, we believe that reducing drug resistance can start from the key nodes of the signaling network and enhance the combination of various therapies. Even in the future, studying epigenetic and chromatin accessibility through new techniques can more effectively predict the underlying mechanisms of emerging drug resistance.
